# The effectiveness of collaborative care delivered via telehealth in a pediatric primary care population

**DOI:** 10.3389/fpsyt.2023.1240902

**Published:** 2023-11-13

**Authors:** Karl Vanderwood, Jian Joyner, Virna Little

**Affiliations:** ^1^JG Research & Evaluation, Bozeman, MT, United States; ^2^Concert Health, San Diego, CA, United States

**Keywords:** depression, anxiety, collaborative care, adolescent mental health, pediatric primary care

## Abstract

**Introduction:**

The prevalence of mental health conditions among children and adolescents in the United States has become a pressing concern, exacerbated by the COVID-19 pandemic. Collaborative care is an evidence-based model for identifying and treating depression and anxiety in healthcare settings, with additional promise for remote healthcare delivery. This study aims to evaluate the impact of a telehealth collaborative care model for adolescents with depression and anxiety in pediatric and primary care settings.

**Methods:**

Secondary analysis was conducted using de-identified national data from Concert Health, a behavioral health medical group offering remote collaborative care across 17 states. Baseline, 90-day, and 120-day assessments of the PHQ-9 and GAD-7 were collected, along with baseline covariates. Stepwise regression analysis was performed to determine the contribution of select covariates to improvement rates.

**Results:**

Among the analyzed data, 263 participants had complete PHQ-9 data, and 230 had complete GAD-7 data. In both the PHQ-9 and GAD-7 groups, over 50% of patients experienced treatment success based on success at discharge, as well as 90- and 120-day improvement rates. Predictors of success at discharge for the GAD-7 group included age at enrollment (OR 1.2258, 95% CI 1.01–1.496), clinical touchpoints (OR 1.1469, 95% CI 1.086–1.218), and lower baseline GAD-7 score (OR 0.9319, 95% CI 0.874–0.992). For the PHQ-9 group, Medicaid was significantly associated with not achieving a 50% reduction in PHQ-9 score at 120 days (OR 0.5874, 95% CI 0.349–0.979).

**Discussion:**

Collaborative care has demonstrated its effectiveness in treating adolescent populations, providing an opportunity to expand access to evidence-based behavioral health treatment for young individuals. Notably, collaborative care is already integrated into the Medicaid fee schedule for 22 states and accepted by all commercial payers. Given that individuals often turn to their trusted primary care providers for behavioral health care, offering collaborative care to adolescents can play a crucial role in addressing the ongoing mental health crisis.

## 1. Introduction

Children and adolescents in the United States have long faced a significant burden of mental health conditions, a concern that has been exacerbated by the COVID-19 pandemic. National data reveal that approximately 16.5% of individuals under the age of 18 experience at least one mental health condition (1), with depression, anxiety, and conduct problems being among the most prevalent (2, 3). Alarmingly, rates of adolescent depression and anxiety have shown a concerning upward trend over time, as indicated by youth health trends between 2016 and 2020, from 3.1% to 4.0% and 7.1% to 9.2%, respectively (4). Recent federally collected data also highlights that young adults aged 18 to 24 are more likely to experience symptoms of depression and anxiety compared to older adults. In 2023, as many as 50% of young adults in this age group reported experiencing such symptoms, compared to approximately one-third of adults overall (5). Providing evidence based interventions, such as collaborative care, in adolescence may play a role in reducing the number of young adults reporting symptoms of depression. Adding to the complexity of this issue is a significant lack of access to care after identification of a behavioral health issue, with nearly half (48.3%) of children and adolescents with mental health conditions not receiving the necessary support (6). This important component of screening and appropriate follow-up care has been recently called out by the US Preventative Services Task Force (7). Nationally there continues to be a workforce shortage for behavioral healthcare providers to meet the growing need for treatment. It is estimated that a third of the population lives in a mental health shortage area and more than half of the counties in the US do not have a psychiatrist (8).

Recognizing the urgency of this situation, leading organizations such as the American Academy of Pediatrics, the American Academy of Child and Adolescent Psychiatry, and the Children’s Hospital Association have declared a national state of emergency in child and adolescent mental health (9). Research shows that the pandemic has amplified the mental health challenges faced by children and adolescents, subjecting them to increased stressors such as fear, worry, and increases in social isolation resulting from disrupted schedules, home confinement, and cancellation of extracurricular activities (10). These concerns are supported by data from widely used screening instruments such as the Patient Health Questionnaire-9 (PHQ-9) (11) and the Generalized Anxiety Disorder-7 (GAD-7) (12), which indicate a significantly higher prevalence of depression and anxiety during the pandemic, with rates of 25.2% and 20.5%, respectively, compared to 11.6% and 12.9% previously reported (13).

Collaborative care is an evidence-based model to identify and treat patients with behavioral health conditions in healthcare settings. Collaborative care was supported by the Center for Medicare and Medicaid Services (CMS) when specific and dedicated codes for collaborative care were approved in 2017, encouraging adoption and supporting a systemic approach to behavioral health populations in primary care. Collaborative care uses a registry for patient tracking and adds a behavioral health provider and psychiatric consultant to the pediatric primary care team. Collaborative care uses a measurement based and treat to target approach to care, seeking a significant reduction in the PHQ-9 or GAD-7 scores in the first 90 days of treatment. Consistent with the collaborative care model patient contacts are based on patient need, often occurring multiple times a week over the course of a monthly care episode.

As the prevalence of adolescent mental health conditions continues to rise, so has the demand for and availability of reliable and accessible evidence-based treatments. Concert Health, a United States based behavioral health medical group providing collaborative care to primary care and pediatric organizations across 17 states has extensively reviewed their care for pediatric patients. Keeping with the fidelity of the collaborative care model Concert Health brings masters level collaborative care managers and either advanced nurse practitioner or physician psychiatric consultants, to deliver behavioral health care via video or telephone consults with patients, as well as providing updates on patient progress directly to primary care providers. Treatment is provided during the course of a monthly episode, often speaking with patients multiple times a week over the course of about 6–8 months of treatment.

Collaborative care is supported by over 90 randomized controlled trials (14–16). This approach has demonstrated effectiveness in reducing symptoms and improving outcomes for pediatric and adolescent populations. At 12-months, collaborative care has been shown to improve depressive symptoms in adolescents receiving primary care, with nearly double the rate of depression response and remission compared to usual care (17). Higher rates of response and remission were also observed in a collaborative care intervention for attention-deficit/hyperactivity disorder (ADHD) and anxiety in pediatric primary care (18). A recent systematic review of trials providing remote collaborative care demonstrated the effectiveness of this model and delivery method, however the results were limited to adult patients (19). The current study contributes to the knowledge base supporting remotely delivered collaborative care outside of the trial setting and by describing the impact of the intervention on adolescent patients.

This study aims to demonstrate the effectiveness of collaborative care for adolescent populations by examining medical records of youth who have received collaborative care as provided by Concert Health, a behavioral health medical group that offers collaborative care via telehealth in pediatric and primary care settings The findings emphasize the importance of considering collaborative care as a means to expand access to evidence-based behavioral health treatment for young individuals.

## 2. Materials and methods

Patient clinical data from the electronic systems of Concert Health were retrieved to evaluate the impact of collaborative care on treatment outcomes. Adolescents who scored over ten on the PHQ-9 or GAD-7 were flagged by healthcare providers for assessment and diagnosis in both healthcare offices and with Concert Health clinicians. Those with PHQ-9 or GAD-7 scores and clinical assessments by primary care providers and care managers during clinic contacts concordant with depression and/or anxiety were included in the analysis. Concert Health delivers collaborative care with fidelity to the evidence-based model, with virtual contact options such as telephone or video contacts being the mode of care. Concert Health ensures fidelity to the model by tracking the core components of Collaborative Care, outcomes with a treat to target approach, registry based population health approach and psychiatric consultation. The treatment choices in the collaborative care model are tailored to individual patient preferences, include medication, talk treatment, symptom monitoring, and goal setting. Concert Health clinicians provide care to patients utilizing one, or multiple, treatment options chosen by the patient. Consistent with the collaborative care model individuals often received multiple contacts per week, especially in the early stages of care, with a contact consisting of an interaction with a clinician lasting more than five minutes. Patients may receive more contacts during initial time in care to optimize engagement and treatment choices. Patient contacts vary depending on treatment choice and patient preference. The monthly case rate for collaborative care payment provides the flexibility for the care managers to be truly patient centered, with contacts as often as clinically necessary and appropriate for the patient. Consistent with the collaborative care model there is no required cadence for contacts, only there be sufficient number of minutes to be reimbursed for the month.

**Figure 1:**
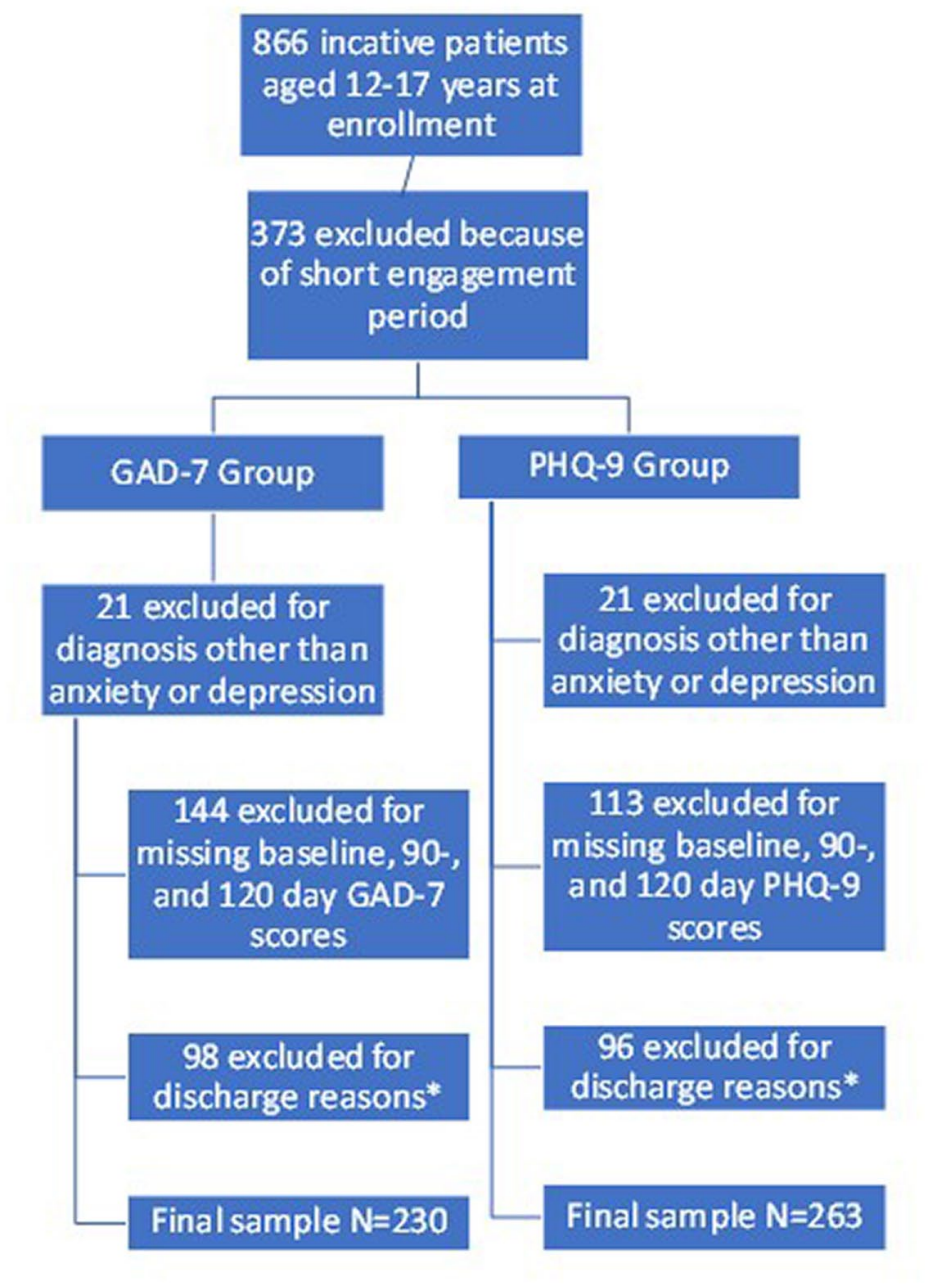
Describes the process used to determine the final analytical sample. * Discharge reasons that led to exclusion included: changed practice/provider, cognitive status, engaged in outside behavioral health center, provider contract ending, referred out for care, referred to community mental health or psychiatric provider.

To be included in the current analysis, patients needed to be inactive, meaning their treatment episode has been completed, between 12 and 17 years old at the time of enrollment, diagnosed with depression and/or anxiety, and have complete baseline, 90-day and 120-day screenings using the PHQ-9 and/or GAD-7 scales. A completed episode is defined as a patient meeting the outcome goal of a score below <10 or < 5 on the PHQ-9 or GAD-7 determined by their baseline score, the patient has met other established treatment goals, or the patient did not meet treatment goals but their time in treatment ended. Patients included in the analysis were enrolled in collaborative care between September 24, 2019, and September 26, 2022. Additional inclusion criteria included documented reasons for discharge, insurance type, program end date, and clinical touchpoint data. Those with multiple inactive treatment episodes during the study period were excluded from the analysis, as were individuals with missing PHQ-9 or GAD-7 scores, and missing data for the independent predictors included in the analysis. Demographic data were limited to age, as gender and race information was not readily available. Figure 1 details the exclusion criteria that result in the final analytical sample for the GAD-7 and PHQ-9 groups. Collaborative care is a primary care model and the CPT codes assigned to Collaborative Care are primary care codes and cover the identification and treatment of adolescents in this project as part of their primary care. Patients engaged in care were referred by their primary care provider and parents or guardians were engaged in the care and treatment, where possible, as that is optimal and a best practice.

Both the GAD-7 and PHQ-9 have been validated to detect anxiety and depression in primary care, respectively (20). In primary care, the GAD-7 has demonstrated high sensitivity and specificity in excess of 0.80 to detect generalized anxiety disorder based on a score of 10 or greater (12). Similarly, a PHQ-9 score > = 10 has shown an 88% sensitivity and specificity to detect major depression among primary care patients (11). These measures provide simple, accessible tools for primary care providers to diagnose and treat two common mental health conditions.

Treatment success was considered through two approaches. First, treatment success was defined as a 50% reduction from baseline in scores on the PHQ-9 and GAD-7 at 90 and 120 days. A 50% reduction in treatment score at 90- and 120 days has been recognized as recommended goal, with which to define improvement before and after treatment (21). In some places, like the state of New York’s Collaborative Care Medicaid Program they encourage a 50% reduction in 70 days. Concert Health uses a similar definition to track improvement over time in GAD-7 scores. Second, patient discharge reasons were categorized as either “successful” or “unsuccessful.” For instance, a patient with a discharge reason indicating “Patient has met treatment goals with a relapse prevention plan” was deemed successful, while a patient with a discharge reason indicating “Disengaged from care” was deemed unsuccessful (Supplementary Table).

An IRB was submitted to Western IRB and determined to be exempt under 45 CFR § 46.104(d) (4).

### 2.1. Statistical analysis

Descriptive statistics, including counts, percentages, and means, were used to summarize demographic characteristics and other independent predictors. The improvement scores at 90 and 120 days were calculated by subtracting the baseline PHQ-9 and GAD-7 scores from the corresponding scores and dividing by the baseline score.

To identify relevant variables associated with the dependent variables, defined as a 50% reduction from baseline in scores on the PHQ-9 and GAD-7 at 90 and 120 days and patient discharge reasons, stepwise logistic regression models were used. The full model included independent variables such as age at enrollment, diagnosis category, insurance type, duration of enrollment, number of touchpoints, and baseline PHQ-9 or GAD-7 scores. A backwards stepwise regression procedure was conducted, initially incorporating all independent variables, and iteratively testing different combinations by adding or removing variables. Odds ratios (ORs) for each variable were calculated and presented with 95% confidence intervals (CIs).

All data analysis was completed using RStudio (22).

## 3. Results

A total of 295 adolescent patients were included in the analysis, with 263 in the PHQ-9 group, 230 in the GAD-7 group, and 198 overlapping in both groups (Table 1). Patients were from Arizona (*n* = 14), California (*n* = 12), Florida (*n* = 1), New Mexico (*n* = 5), New York (*n* = 262), and Texas (*n* = 1) and received care at family practice (*n* = 14), internal medicine (*n* = 1), multi-specialty (*n* = 18), obstetrics and gynecology (*n* = 1), pediatrics (*n* = 4) and other/unknown (*n* = 257) most of which are small independent and/or private practice primary care sites It is important to note that New York, where the majority of patients are located in the current study, has a dedicated collaborative care Medicaid program and was one of the first states to include collaborative care on the Medicaid fee schedule. For the PHQ-9 group, a higher proportion were diagnosed with depressive disorder (64.3%), had Medicaid insurance (53.2%), and had a mean baseline PHQ-9 score of 10.6 (SD 5.49). In the GAD-7 group, the majority had Medicaid insurance (54.8%) and had a mean baseline GAD-7 score of 8.41 (SD 4.94). Both the PHQ-9 and GAD-7 groups had similar mean durations of enrollment and number of clinical touchpoints (Table 1).

**Table 1:** Patient demographics by group.

	GAD-7 group (*N* = 230)	PHQ-9 group (*N* = 263)
Age group		
12 years	19 (8.3%)	23 (8.7%)
13 years	30 (13.2%)	30 (11.4%)
14 years	43 (18.7%)	52 (19.8%)
15 years	44 (19.1%)	51 (19.4%)
16 years	43 (18.7%)	53 (20.2%)
17 years	51 (22.2%)	54 (20.5%)
Diagnosis category		
Anxiety disorder	114 (49.6%)	94 (35.7%)
Depressive disorder	116 (50.4%)	169 (64.3%)
Insurance type		
Commercial	104 (45.2%)	123 (46.8%)
Medicaid	126 (54.8%)	140 (53.2%)
Days enrolled		
Mean (SD)	200 (107)	205 (106)
Median [min, max]	169 [90.0, 647]	173 [90.0, 647]
Clinical touchpoints		
Mean (SD)	10.9 (7.34)	11.2 (7.5)
Median [min, max]	9.0 [0, 43.0]	10.0 [0, 43.0]
Baseline Assessment Score		
Mean (SD)	8.41 (4.94)	10.6 (5.49)
Median [min, max]	8.5 [0, 19.0]	10.0 [0, 26.3]

Compared to adolescents excluded from the analysis (*n* = 571), those included in the analysis were older [14.9 (SD 1.6) vs. 14.6 (SD 1.6) years, *p* = 0.02], a higher proportion were diagnosed with depressive disorder (58.6% vs. 40.1%), a higher proportion had commercial insurance (47.8% vs. 40.8%), and fewer had Medicaid insurance (52.2% vs. 58.0%), Baseline assessment scores were similar between those included and excluded from the analysis, with GAD-7 baseline scores of 8.41 (SD 4.94) vs. 8.78 (SD5.26) (*p* = 0.38), and PHQ-9 baseline scores of 10.6 (SD5.49) vs. 10.2 (SD6.29) (*p* = 0.36), among those included and excluded from the analysis, respectively (data not shown).

### 3.1. Determining treatment success

Among patients in the PHQ-9 group, 70.7% achieved a successful treatment episode at discharge as evidenced by the stated discharge reason. At 90 days, 58.9% of the PHQ-9 group had a 50% reduction in PHQ-9 score, while at 120 days, this percentage increased to 62.4%. In the GAD-7 group, 67.4% of patients had a successful treatment episode based on the discharge reason, with 50.9% achieving a 50% reduction in GAD-7 score at 90 days and 53.5% at 120 days (Table 2).

**Table 2:** Treatment success determined by discharge reason and 50% reduction in score at 90 and 120 days by group.

Success metric	Unsuccessful	Successful
	GAD-7 group (*N* = 230)	
Success at discharge	75 (32.6%)	155 (67.4%)
50% reduction at 90 days	113 (49.1%)	117 (50.9%)
50% reduction at 120 days	107 (46.5%)	123 (53.5%)
	PHQ-9 group (*N* = 263)	
Success at discharge	77 (29.3%)	186 (70.7%)
50% reduction at 90 days	108 (41.1%)	155 (58.9%)
50% reduction at 120 days	99 (37.6%)	164 (62.4%)

In the GAD-7 group, there were no significant associations between the independent variables and a 50% reduction in GAD-7 score at 90 or 120 days based on backwards stepwise regression modelling (data not shown). However, successful completion of the treatment episode at discharge was significantly associated with increasing age (*p* < 0.05), a greater number of clinical touchpoints (*p* < 0.001), and lower baseline GAD-7 scores (*p* < 0.05) (Table 3).

**Table 3:** Results of stepwise logistic regression and success at discharge among the GAD-7 group.

	Outcome: success at discharge = 1		
Category	Variable	Odds ratio	95% CI
	Intercept	0.0373	0.002–0.661
	Age at enrollment	1.2258	1.010–1.496*
Insurance type	Medicaid	1.6524	0.898–3.060
	Clinical touchpoints	1.1469	1.086–1.218***
	GAD-7 baseline score	0.9319	0.874–0.992*

All patients with incomplete data were removed from the data. Insurance type is a categorical variable with commercial insurance as the reference group. Statistical significance levels: *** < 0.001, ** < 0.01, * < 0.05.

Regarding the PHQ-9 group, achieving a 50% reduction in PHQ-9 score at 120 days was significantly associated with insurance type. Patients enrolled in Medicaid were less likely (OR = 0.5874 95% CI 0.349–0.979, *p* < 0.005) to achieve a 50% reduction compared to patients enrolled in commercial insurance (Table 4). There were no significant associations between the independent variables and a 50% reduction in PHQ-9 score at 90 days (data not shown). Successful completion of the treatment episode at discharge was associated with a higher number of clinical touchpoints (OR = 1.1543 95% CI 1.095–1.224, *p* < 0.001) among the PHQ-9 group (data not shown).

**Table 4:** Results of stepwise logistic regression and 50% improvement at 120 days among the PHQ-9 group.

	Outcome: 50% improvement at 120 days = 1		
Category	Variable	Odds ratio	95% CI
	Intercept	2.7121	1.394–5.395***
Insurance type	Medicaid	0.5874	0.349–0.979**
Diagnosis category	Depressive disorder	1.5618	0.914–2.678
	Days enrolled	0.9977	0.995–1.000*

All patients with incomplete data were removed from the data. Diagnosis category and insurance type area categorical variables. The reference group for insurance type is commercial insurance and the reference group for diagnosis category is anxiety. Statistical significance levels: *** < 0.01, ** < 0.05, * < 0.1.

## 4. Discussion

Expansion of the collaborative care model holds significant potential for improving access to evidence-based behavioral health treatment for diverse patient populations and communities nationwide. The patient-centered nature of collaborative care, where the patient and their treatment goals are central to therapeutic relationship of their primary care provider and behavioral health consultants, has been shown to yield better outcomes compared to traditional mental health treatments. This model allows for more frequent “doses” of treatment, providing the additional support and reinforcement that many children and adolescents require. The monthly case rate billing and optimizing remote communications allow for the flexibility to contact patients in more frequent, shorter contacts to reinforce goals or skills and provide support. The results of this study further strengthen the association between success at discharge and the number of clinical touchpoints or “doses” among patients in the GAD-7 group. Moreover, collaborative care demonstrates significantly higher rates of successful outcomes and treatment completion compared to traditional community mental health or embedded behavioral health treatment approaches (15, 23). Although the number of clinical touchpoints were significantly associated with successful treatment outcomes in the GAD-7 group, future research should consider the effects of both the duration of the treatment episode and the cadence of the clinical touchpoints on treatment outcomes.

In this study, more than half of the adolescent patients enrolled in collaborative care for depression and anxiety achieved treatment success, as indicated by reduced assessment scores or discharge reasons. The results of the current study are similar (24) or more promising (25, 26) than other forms of remotely delivered interventions to treat depression. It should be noted that all of these studies were conducted among adults and the outcome measure differed slightly than the current study in that it included a 50% reduction in PHQ-9 score and a PHQ-9 score < 10 (24–26). The current findings provide support for the effectiveness of evidence-based collaborative care and emphasize the importance of implementing such a system of care for clinicians addressing adolescents with depression and anxiety. Future research should explore a more comprehensive evaluation of treatment characteristics to further understand treatment improvement, in addition to changes in assessment scores as was done in the current study. Potential areas of inquiry may include the length and frequency of clinical touchpoints, medication adherence, and longitudinal follow-up to identify additional treatment episodes to address behavioral health needs.

Furthermore, the results of this study indicate strong evidence for the effectiveness of collaborative care in supporting adolescent populations using validated tools such as the PHQ-9 and GAD-7, which are suitable for ages 12 and up. The study reveals a positive association between age and the likelihood of improved outcomes among patients treated for anxiety, suggesting that as adolescents mature, their chances of experiencing better results increase. This association can be attributed in part to older adolescents’ greater capacity to actively engage in the predominantly telephonic, short-term, symptom-based activities and treatment choices offered in collaborative care. Additionally, some of the helpful skills like progressive relaxation, mindfulness or breathing may be more easily learned and adopted with increased age. Future investigations of collaborative care among adolescents should seek to include the amount of parental involvement that occurs over the course of a treatment episode to identify if a relationship exists between the age of the patient and the amount of assistance that is required form parents or guardians.

Although the relationship between Medicaid enrollment and treatment success only reached significance in one model, this result is worth noting. Patients in the PHQ-9 group enrolled in Medicaid were nearly 42% less likely to achieve a 50% reduction in PHQ-9 scores at 120 days compared to patients with commercial insurance. A study comparing mental health treatment outcomes among adolescent and young adult patients with public and private insurance found similar reductions in depression, suicidal ideation, and non-suicidal self-injury across groups (27). Patients enrolled in Medicaid are often face additional barriers to treatment engagement, however little research exists to describing the effect of collaborative care on behavioral health outcomes among this group. The contradictory findings of the current study, suggesting poorer outcomes among Medicaid patients, and the dearth of currently available research warrant further investigation to identify whether additional support needs to be provided and if this relationship holds true across other adolescent populations. Important aspects to consider in future implementation of collaborative care delivered via telehealth are barriers related to socioeconomic status and technology that may disproportionately affect low-income populations that rely on Medicaid (28).

The declaration of a children’s mental health crisis has brought significant attention to the urgent need for improved access to behavioral health treatment for children and adolescents, especially given that many of them live in areas with limited availability of child psychiatric providers (29). Additionally, many children and adolescents initially seek behavioral health care from their trusted pediatric primary care providers rather than community mental health services (30). Therefore, it is crucial for these providers to have systems in place for early identification and treatment, fostering prevention and ensuring that patients with greater needs have improved access to specialty care.

### 4.1. Limitations

There are limitations to consider in the current study. First, the absence of data on patients’ gender and race/ethnicity prevented investigation of the impact of these variables on treatment outcomes. This prevented the authors from disaggregating the data and limited the ability of the current study to provide insight regarding the impact of collaborative care on the advancement of health equity. Second, due to missing information for some independent variables and short engagement periods reducing the number of patients completing 120-day assessments, the sample size in the analysis was limited. Although patients are generally more engaged in collaborative care in comparison to more traditional care modalities, many patients will often discontinue care when they start to “feel better” as opposed to continuing for optimal treatment results as evidenced by 90- and 120-day assessment scores. Third, no control group was included in this study to compare other treatment modalities to collaborative care.

### 4.2. Conclusion

This study provides compelling evidence that collaborative care is an effective approach to addressing depression and anxiety in adolescents. These findings can inform and encourage the adoption of collaborative care by healthcare providers and organizations, thereby leading to improved access to evidence-based behavioral health treatment for children and adolescents within their communities and in coordination with their healthcare providers. This integrated approach facilitates true comprehensive, patient-centered care.

## Data availability statement

The original contributions presented in the study are included in the article/Supplementary materials, further inquiries can be directed to the corresponding author.

## Ethics statement

The studies involving humans were approved by Western Institutional Review Board. The studies were conducted in accordance with the local legislation and institutional requirements. Written informed consent for participation in this study was provided by the participants’ legal guardians/next of kin. Written informed consent was obtained from the minor(s)’ legal guardian/next of kin for the publication of any potentially identifiable images or data included in this article.

## Author contributions

VL, JJ, and KV were involved in the conception and design of the study and contributed to the revision process, provided critical feedback, edited the manuscript. VL and JJ took the lead in drafting the initial version of the manuscript. KV conducted the data analysis and contributed to writing specific sections of the manuscript. All authors contributed to the article and approved the submitted version.
