# Supplementary material for: The effectiveness of collaborative care delivered via telehealth in a pediatric primary care population
Source: Front Psychiatry. 2023 Nov 13;14:1240902. doi: 10.3389/fpsyt.2023.1240902 (PMC10679399; doi:10.3389/fpsyt.2023.1240902)
Supplement: Supplementary file 1 [file Table_1.docx]

Supplementary Table. Rationale for determining “success” at discharge.

| **Discharge reason** | **Successful discharge (Y/N)** | **Rationale for determining success** |
| --- | --- | --- |
| Declined treatment | N | Not successful because patient declined treatment |
| Disengaged from care | N | Not successful because patient disengaged from care |
| Healthy Days - Met goals, achieved 4 | Y | Successful because patient met treatment goals |
| Healthy Days - Met goals, reduction of 4 or more | Y | Successful because patient met treatment goals |
| Met treatment goals | Y | Successful because patient met treatment goals |
| Non-responsive | N | Not successful because patient did not respond |
| Other | N | Not successful because no information provided regarding patient status at discharge |
| Patient declined due to consent issue | N | Not successful because patient did not participate in care |
| Patient disengaged from treatment with reduced survey score | N | Not successful because patient disengaged from care |
| Patient has met treatment goals with a relapse prevention plan | Y | Successful because patient met treatment goals |
| Patient has met treatment goals without a relapse prevention plan | Y | Successful because patient met treatment goals |
| Refused service | N | Not successful because patient did not participate in care |
